# Polyhedrocytes in blood clots of type 2 diabetic patients with high cardiovascular risk: association with glycemia, oxidative stress and platelet activation

**DOI:** 10.1186/s12933-018-0789-6

**Published:** 2018-11-22

**Authors:** Grzegorz Gajos, Aleksander Siniarski, Joanna Natorska, Michał Ząbczyk, Jakub Siudut, Krzysztof Piotr Malinowski, Renata Gołębiowska-Wiatrak, Paweł Rostoff, Anetta Undas

**Affiliations:** 1 Department of Coronary Artery Disease and Heart Failure, Jagiellonian University Medical College, Prądnicka 80 St., 31-202 Kraków, Poland; 2 Institute of Cardiology, Jagiellonian University Medical College, Prądnicka 80 St., Kraków, Poland; 3 John Paul II Hospital, Prądnicka 80 St., Kraków, Poland; 4 Institute of Public Health, Faculty of Health Sciences, Jagiellonian University Medical College, Grzegórzecka 20 St., Kraków, Poland

**Keywords:** Diabetes, Fibrin, Blood clot, Polyhedrocytes, Protein carbonylation

## Abstract

**Background:**

Little is known about factors that affect the composition of contracted blood clots in specific diseases. We investigated the content of polyhedral erythrocytes (polyhedrocytes) formed in blood clots and its determinants in type 2 diabetes (T2D) patients.

**Methods:**

In 97 patients with long-standing T2D [median HbA_1c_, 6.4% (interquartile range 5.9–7.8)], we measured in vitro the composition of blood clots, including a clot area covered by polyhedrocytes using scanning electron microscopy and the erythrocyte compression index (ECI), defined as a ratio of the mean polyhedrocyte area to the mean native erythrocyte area. Moreover, plasma fibrin clot permeability (K_s_), clot lysis time (CLT), thrombin generation, oxidative stress [total protein carbonyl (total PC), total antioxidant capacity and thiobarbituric acid reactive substances (TBARS)], and platelet activation markers were determined. The impact of glucose concentration on polyhedrocytes formation was assessed in vitro.

**Results:**

Polyhedrocytes content in contracted clots was positively correlated with glucose (r = 0.24, p = 0.028), glycated hemoglobin (r = 0.40, p = 0.024), total cholesterol (r = 0.22, p = 0.044), TBARS (r = 0.60, p = 0.0027), P-selectin (r = 0.54, p = 0.0078) and platelet factor-4, PF4 (r = 0.59, p = 0.0032), but not with thrombin generation, platelet count, K_s_ or CLT. Patients who formed more polyhedrocytes (≥ 10th percentile) (n = 83, 85.6%) had higher glucose (+ 15.7%, p = 0.018), fibrinogen (+ 16.6%, p = 0.004), lower red blood cell distribution width (RDW, − 8.8%, p = 0.034), reduced plasma clot density (− 21.8% K_s_, p = 0.011) and impaired fibrinolysis (+ 6.5% CLT, p = 0.037) when compared to patients with lesser amount of polyhedrocytes (< 10th percentile). ECI and the content of polyhedrocytes were strongly associated with total PC (r = 0.79, p = 0.036 and r = 0.67, p = 0.0004, respectively). In vitro an increase of glucose concentration by 10 mmol/L was associated with 94% higher polyhedrocytes content (p = 0.033) when compared to the baseline (7.1 mM). After adjustment for age, sex and fibrinogen, multiple regression analysis showed that RDW was the only independent predictor of polyhedrocytes content in T2D (OR = 0.61, 95% CI 0.39–0.92).

**Conclusions:**

Poor glycemic control, together with enhanced platelet activation and oxidative stress, increase the content of polyhedrocytes in blood clots generated in T2D patients.

**Electronic supplementary material:**

The online version of this article (10.1186/s12933-018-0789-6) contains supplementary material, which is available to authorized users.

## Introduction

Type 2 diabetes (T2D) is associated with a hypercoagulable state involving increased platelet activation, thrombin generation and unfavorable fibrin clot properties [1–4]. T2D patients are at high risk of arterial thromboembolism [1, 2, 5] and associated mortality [6–9]. T2D patients present significant changes in the morphology of their erythrocytes and in the nature of the fibrin formed upon the addition of thrombin [10]. Platelet count is usually within the reference range, however platelet activity is increased [11] as reflected among others by elevated platelet factor 4 or P-selectin in T2D patients [12–14]. Moreover, platelet contractile force has been shown to be increased in T2D patients [15]. During clot formation platelets activated by thrombin form a platelet–fibrin mesh constituting the basic structure of thrombus in vivo or whole blood clot in vitro [16]. Such clots undergo volume shrinkage [17] that is called clot contraction or retraction [18]. Cellular blood components, in particular red blood cells (RBCs), platelets, and plasma fibrinogen concentration influence the rate and extent of clot contraction [18]. It has been postulated that RBCs enhance functional coagulation properties and platelet aggregation. RBC aggregation and decreased deformability are the dominant hematological abnormalities in T2D subjects and may lead to the development of microvascular complications [19]. Reduced clot contraction has been demonstrated in subjects with a lower platelet count and/or dysfunction, elevated hematocrit, leukocytosis, increased plasma fibrinogen, and other changes in blood composition that may affect platelet function and properties of blood clots [20] [21]. It has been suggested that defective clot retraction contributes to arterial and venous thrombosis [22]. Cines et al. have demonstrated that contracted blood clots and arterial thrombi developed a tessellated structure of polyhedral erythrocytes, called polyhedrocytes [18]. Polyhedrocytes were observed in the interior of the blood clot, where the RBCs were tightly compressed, with meshwork of fibrin and platelets on its surface [18, 23]. It has been postulated that polyhedrocytes provide impermeable seal, due to minimal interstitial space, which stems bleeding and promotes fibrinolysis resistance [18, 23]. We have reported that polyhedrocytes are observed in 20% of thrombi obtained from epicardial arteries of ST-elevation myocardial infarction patients [18, 23, 24]. Formation of polyhedrocytes in arterial thrombi has been associated with higher RBC count and both lower platelet count and plasma fibrinogen, but not with cardiovascular risk factors or the ischemia time [25].

To our knowledge, there have been no reports on determinants of polyhedrocyte formation in clots of patients with T2D. RBCs are extremely sensitive to oxidative stress or cytokine upregulation. This usually accompanies systemic inflammation in most diseases, including T2D [26]. We hypothesized that poor metabolic control, along with prothrombotic plasma clot phenotype, enhanced platelet activation and oxidative stress observed in T2D patients with high cardiovascular risk, are associated with higher content of polyhedrocytes.

## Research design and methods

We enrolled 97 consecutive T2D patients with high cardiovascular risk hospitalized from August 2015 to April 2016 in John Paul II Hospital in Krakow, Poland and 30 apparently healthy controls. All patients fulfilled the American Diabetes Association criteria for diagnosis of T2D and were treated for at least 12 months. The exclusion criteria were arterial or venous thromboembolic events within previous 6 months, current anticoagulant therapy, known cancer, signs of acute infection, chronic inflammatory disorders (e.g. rheumatoid arthritis), liver injury (alanine or asparagine transaminase > 1.5 times above the upper limit of the reference range), estimated glomerular filtration rate (eGFR) < 30 mL/min, and pregnancy. Current smoking was defined as smoking at least one cigarette daily. Arterial hypertension was defined as a systolic and/or diastolic blood pressure of ≥ 140 mmHg or ≥ 90 mmHg, respectively or a history of arterial hypertension with or without taking antihypertensive agents. Coronary artery disease (CAD) was established based on a documented history of myocardial infarction (MI) or a positive result of ECG stress test or gated single photon emission computed tomography with Tc-99 m-MIBI (SPECT) or a coronary angiography. Previous MI was established based on medical records.

The Jagiellonian University Ethical Committee approved the study and all the participants provided their written informed consent.

### Routine laboratory investigations

Fasting venous blood was drawn between 7 and 10 A.M. and was kept at a room temperature. Blood samples were collected into citrated tubes (9:1 of 0.106 M sodium citrate), centrifuged at 2500*g* and 20 °C for 10 min to obtain platelet poor plasma (PPP), snap-frozen within 60 min, and stored in small aliquots at − 80 °C until analysis. The activated partial thromboplastin time (aPTT), creatinine, eGFR, serum total cholesterol (TC), low-density lipoprotein cholesterol (LDL-C), high-density lipoprotein cholesterol (HDL-C), triglycerides (TG), glucose and thyroid stimulating hormone (TSH) were assayed by routine laboratory techniques. Glycated hemoglobin A1c (HbA_1c_) was measured using immunoturbidimetry (Roche Diagnostics GmbH, Mannheim, Germany). Complete blood count including white blood cells (WBC), RBC, hemoglobin, hematocrit, red blood cell distribution width (RDW), platelet count and platelet distribution width were assayed. Fibrinogen was assessed using the Clauss method. The hsCRP was determined using immunoturbidimetry (Roche Diagnostics GmbH, Mannheim, Germany). Immunoenzymatic assay was used to determine plasminogen activator inhibitor-1 (PAI-1) antigen (American Diagnostica, Stamford, CT, USA) in citrated plasma. Plasma α2-antiplasmin (α2AP) and plasminogen were measured by chromogenic assays (STA Stachrom antiplasmin and STA Stachrom plasminogen, Diagnostica Stago, Asnieres, France). Markers of platelet activation, P-selectin (CD62P) and platelet factor-4 (PF4), were determined in citrated plasma by ELISAs (R&D Systems, Abington, UK). The interassay and intraassay coefficients of variation for all the ELISAs were < 8%.

### Preparation of whole blood clots

Clotting was initiated by addition of 2 µL of activation mixture (CaCl_2_ [Sigma-Aldrich, St. Louis, MO, US] and human thrombin [Merck KGaA, Darmstadt, Germany] at final concentrations of 0.01 M and 1 U/mL, respectively) to 48 μL of whole blood from the antecubital vein that was prewarmed for 5 min at 37 °C. The samples were incubated at room temperature for 24 h.

### Scanning electron microscopy

**Fig. 1:**
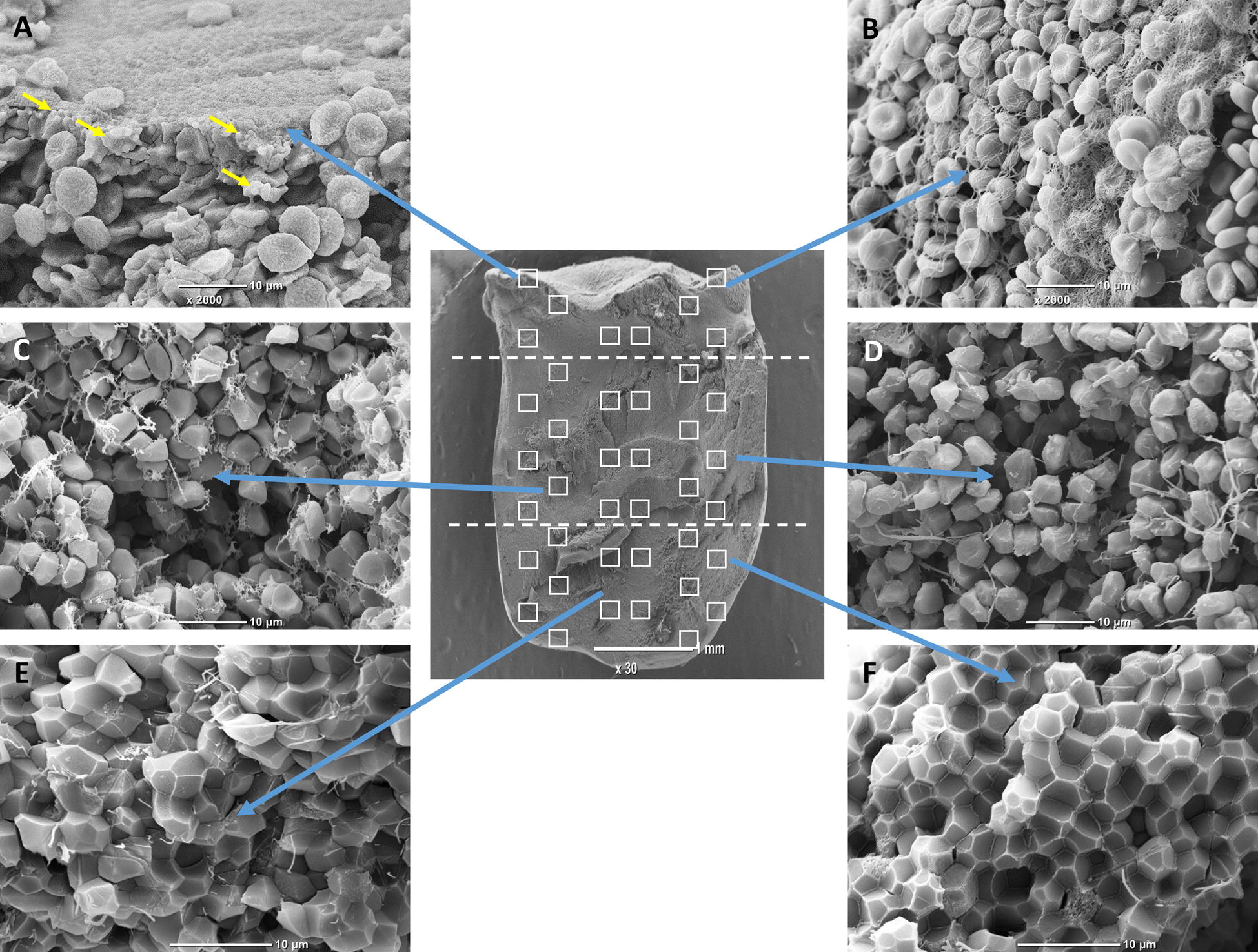
A representative SEM images of a retracted whole blood clot (magnifications ×30 and ×3600) used for semiquantitative analysis of polyhedrocytes content and size measurement in 40 selected areas located in 3 vertical axes (from the left to the right and from the top to the bottom of the clot). **a** and **b** clot surface area containing fibrin cup and native red blood cells (RBCs) and eryptotic cells (marked with arrows), **c** and **d** transitional clot area composed mostly of transitional forms of polyhedrocytes and fibrin fibers, **e** and **f** clot internal area mainly composed of polyhedrocytes and small amounts of fibrin located on the edges of polyhedral RBCs

Scanning electron microscope (SEM) analysis was performed as previously described [27]. Blood clots were washed in 0.1 M NaCl for several minutes and fixed in 2.5% glutaraldehyde, dehydrated in raised ethanol concentrations and frozen in tert-Butyl alcohol for 2 h. Then, clots were dried in a vacuum and coated with gold. High-definition photographs were acquired using a scanning electron microscope (JEOL JCM-6000, Japan). We performed analysis of polyhedrocytes content and size measurement in 40 selected areas located in 3 vertical axes (from the left to the right and from the top to the bottom of the clot) with a subjective evaluation of the area covered by RBC, polyhedrocytes, or their transitional forms (Fig. 1).

For the semiquantitative analysis, each picture was divided into 400 squares and the dominant composition (platelets, fibrin, erythrocytes, WBC) of each was assayed (Fig. 1). The visual approach consisted of a careful observation of each pictures with a subjective evaluation of the percentage of each component. Two independent investigators performed visual approach. Very low interindividual variability was found in the clot composition analysis (< 7%). The investigators performing SEM and analysis were blinded to the clinical data. Images for each clot were then assessed using ImageJ (US National Institutes of Health) to determine the area (µm^2^) of native RBCs and polyhedrocytes. Results were presented as means ± standard deviations (SD) of about 100 consecutive RBC areas obtained from each SEM image.

### Erythrocyte compression index

Erythrocyte compression index (ECI) was determined as previously described [28]. Briefly, ECI is defined as a ratio of the mean polyhedrocyte area to the mean native RBC area expressed as a percentage. Two investigators evaluated the first 10 clots with the inter-observer and intra-observer agreement of 93% and 95%, respectively.

### Thrombin generation potential

Plasma thrombogenic potential was assessed using calibrated automated thrombography (CAT) (Thrombinoscope BV, Maastricht, the Netherlands) in a 96-well plate fluorometer (Ascent Reader, Thermolab systems OY, Helsinki, Finland) at 37 °C according to the manufacturer’s instructions. Eighty microliters of platelet poor plasma were diluted with 20 µL of a commercially available tissue factor (TF)-based activator (Diagnostica Stago, Asniéres, France) containing 5 pM recombinant TF, 4 micromolar phosphatidylserine/phosphatidylcholine/phosphatidylethanolamine vesicles, and 20 µL of FluCa solution (Hepes, pH 7.35, 100 nmol/L CaCl_2_, 60 mg/mL bovine albumin, and 2.5 mmol/L Z-Gly-Gly-Arg-amidometylcoumarin). The reference ranges of thrombin generation potential were as follows: Lag time, 1.57 ± 0.33 min; ETP, 1694 ± 373 nM × min; Peak thrombin generation, 360 ± 108 nM; ttPeak, 3.96 ± 0.71 min [29].

### Fibrin clot permeability and lysis

Fibrin clot permeability was determined using a pressure-driven system [30]. Briefly, 20 mM calcium chloride and 1 U/mL human thrombin (Merck KGaA) were added to 120 µL citrated plasma. A permeation coefficient (K_s_), which indicates the pore size, was calculated from the equation:

$${\text{K}}_{\text{s}} = {\text{Q}} \times {\text{L}} \times \,\upeta/{\text{t}} \times {\text{A}} \times \Delta {\text{p}},$$

where Q is the flow rate in time t; L, the length of a fibrin gel; η, the viscosity of liquid (in poise); A, the cross-sectional area (in cm^2^), and Δp, a differential pressure (in dyne/cm^2^).

To assess efficiency of clot lysis, citrated plasma was mixed with 15 mM calcium chloride, 6 pM human tissue factor (Innovin, Siemens), 12 μM phospholipid vesicles and 60 ng/mL recombinant tPA (rtPA, Boehringer Ingelheim, Germany). The mixture was transferred to a microtiter plate and its turbidity was measured at 405 nm at 37 °C. Clot lysis time (CLT) was defined as the time from the midpoint of the clear-to-maximum-turbid transition, which represents clot formation, to the midpoint of the maximum-turbid-to-clear transition (representing the lysis of the clot). The reference ranges of fibrin clot permeability and lysis were as follows: Ks, 7.01 ± 2.06 × 10^−9^cm^2^; CLT, 100 ± 9.7 min [29, 31].

### Total protein carbonyl (total PC) assessment

Oxidative modification of plasma proteins was assessed based on carbonyl content using 2–4 dinitrophenylhydrazine (DNPH), as reported by Becatti et al. [32]. DNPH reacts with PC, forming a Schiff base to produce the corresponding hydrazone, which can be analyzed spectrophotometrically. Briefly, citrated PPP (100 µL) after incubation with DNPH (400 µL) was precipitated with trichloracetic acid (TCA) and the pellet washed several times with a 1:1 mixture of ethanol/ethyl acetate. Finally, the pellet was resuspended in 500 µL guanidine hydrochloride and measured at 370 nm. PC content was calculated by using a molar extinction coefficient of 22,000 M^−1^ cm^−1^. The results, expressed as nM/mL of PC, were then normalized for protein concentration. The reference range of total PC was 1.82 ± 0.2 nmol/mg [33].

### TBARS (thiobarbituric acid reactive substances) estimation

Thiobarbituric acid reactive substances levels were measured in citrated PPP using a TBARS assay kit (OXI-TEK, ENZO, USA) in accordance with the manufacturer’s instructions as previously reported [32]. Briefly, the adduct generated by reacting malondialdehyde with thiobarbituric acid after 1 h at 95 °C was measured spectrofluorimetrically, with excitation at 530 nm and emission at 550 nm. TBARS were expressed in terms of malondialdehyde equivalent (nM/mL) and then normalized for protein concentration. The reference range of TBARS was: 18.6 ± 1.16 nmol/mL [33].

### Total antioxidant capacity (TAC) assay

The ORAC method (oxygen radical absorbance capacity), based on the inhibition of the peroxyl-radical-induced oxidation initiated by thermal decomposition of azo-compounds, like 2,2icazobis(2-amidinopropane) dihydrochloride (AAPH), was performed as reported by Becatti et al. [32]. Briefly, a fluorescein solution (6 nM) prepared daily from a 4 µM stock in 75 mM sodium phosphate buffer (pH 7.4), was used. Trolox (250 μM final concentration) was used as a standard. 70 μL of citrated PPP with 100 μL of fluorescein were pre-incubated for 30 min at 37 °C in each well, before rapidly adding AAPH solution (19 mM final concentration). Fluorescence was measured with excitation at 485 nm and emission at 537 nm in a Fluoroskan Ascent Microplate Fluorometer (Thermo Fisher Scientific Inc. MA, USA). Results were expressed as Trolox Equivalents (μM) and then normalized for protein concentration. The reference range of TAC assay was: 378.86 ± 20.82 nmol/mL [33].

The oxidation parameters, platelet markers, and fibrinolytic proteins were measured in a subset of patients (n = 23).

### An influence of exogenous glucose on polyhedrocyte formation

To assess the direct influence of glucose concentration on polyhedrocytes formation, we added 10 mM glucose solution in 0.9% sodium chloride to blood obtained from randomly selected 10 healthy (5 men and 5 women aged 39–60 [median, 47] years; mean glucose level of 4.9 [IQR: 4.6–5.2] mM) and 7 randomly selected T2D subjects (3 men and 4 women aged 31–76 [median, 74] years; mean glucose level of 7.1 [IQR: 6.6–7.9] mM). The samples were incubated at 37 °C for 1 h. Then, the whole blood clots were prepared in duplicate and analyzed as described above by two independent investigators blinded to the clinical data.

### Statistical analysis

Continuous variables were presented as mean and standard deviation or median with the first and the third quartile depending on data distribution assessed using Shapiro–Wilk test and compared using Student’s t test or Wilcoxon test. Nominal variables were described using counts and percentages and compared using χ^2^ test of Fisher’s Exact Test. Pearson’s or/and Spearman’s correlations were calculated when applicable for measuring the association between two continuous variables. The change in analyzed variables due to the increase in polyhedrocytes by 1% was assessed using linear regression. Several logistic regression models with production of polyhedrocytes as a dependent variable (production occurred or not occurred). The best model was then obtained stepwise backwards minimalizing Bayesian Information Criterion (BIC) with variables gender, age and level of fibrinogen locked in the model for adjustment. Association between the variables was expressed as odds ratio (OR) with corresponding 95% confidence intervals (CI). A p-value < 0.05 was considered statistically significant.

## Results

### Patient characteristics

The final analysis included 97 patients with a well-controlled (median HbA_1c_, 6.4%), long-standing (median duration, 9.3 years) T2D (Table 1) and 30 age-matched healthy controls (64.39 ± 10.12 years) not taking any medications. A majority of patients were at high risk of CAD and were treated as recommended in the current guidelines, including aspirin (Table 1).

**Table 1:** Patient characteristics

Variable	All patients (n = 97)	Low polyhedrocytes group (n = 14)	High polyhedrocytes group (n = 83)	p-value
Male sex, n (%)	63 (64.95)	11 (78.57)	52 (62.65)	0.37
Age, years	67.49 ± 8.82	70.64 ± 10.98	66.96 ± 8.36	0.15
Current smoking, n (%)	22 (22.68)	4 (28.57)	18 (21.69)	0.73
BMI, kg/m^2^	30.5 (27.4–34.4)	30.4 (28.1–35.7)	30.6 (27.2–34.1)	0.79
Hypertension, n (%)	93 (95.88)	13 (92.86)	80 (96.39)	0.47
Coronary artery disease, n (%)	74 (76.29)	11 (78.57)	63 (75.90)	1.00
T2D time since diagnosis, years	9.30 (5.75–14.30)	7.50 (5.25–15.00)	9.45 (6.00–13.98)	0.94
Drugs				
Sulphonylurea, n (%)	37 (38)	7 (50)	30 (36)	0.38
Biguanide, n (%)	61 (63)	6 (43)	55 (66)	0.13
Insulin, n (%)	25 (26)	4 (29)	21 (25)	0.75
Beta-blocker, n (%)	82 (85)	12 (86)	70 (84)	1.00
ACEI, n (%)	67 (69)	12 (86)	55 (66)	0.21
Calcium antagonist, n (%)	32 (33)	3 (21)	29 (35)	0.38
Clopidogrel, n (%)	22 (23)	5 (36)	17 (20)	0.30
Aspirin, n (%)	79 (81)	13 (93)	66 (80)	0.46
Statin, n (%)	80 (82)	14 (100)	66 (80)	0.12
ARB, n (%)	17 (18)	2 (14)	15 (18)	1.00
Loop diuretic, n (%)	30 (31)	6 (43)	24 (29)	0.35
Spironolactone, n (%)	22 (23)	3 (21)	19 (23)	1.00

Values are given as number (percentage), mean ± SD or median (interquartile range)

Low polyhedrocytes group represents lowest 10% of the observations. High polyhedrocytes group represents highest 90% of the observations

*ACEI* angiotensin-converting-enzyme inhibitors, *ARB* angiotensin receptor blockers, *BMI* body mass index, *MI* myocardial infarction, *T2D* type 2 diabetes

### Polyhedrocytes formation

We divided the study group based on the percentage of the polyhedrocytes content on the surface of contracted blood clots. Patients in the lowest polyhedrocyte group (below 10th percentile of the observations, n = 14) were considered as a low-polyhedrocytes group (LP), in contrast to the rest of the patients (latter 90% of observations, n = 83) who were considered as a high-polyhedrocytes group (HP). Median percentage of polyhedrocytes in blood clots in the HP group was 20% (interquartile range, IQR, 7.7–32.5%) and it was 51% lower than in healthy controls (41 [30–61.5]%, p < 0.01). There were no differences between the HP and LP groups in demographic and clinical characteristics including the medications used (Table 1). The HP group had higher glucose and fibrinogen levels (+ 15.7%, p = 0.018; + 16.6%, p = 0.004 respectively) and lower RDW (− 8.8%, p = 0.034) with no difference in leukocyte or platelet count and lipid profiles (Table 2), when compared with the LP subjects. Additionally, the HP group showed lower Ks by 21.8% (p = 0.011) and increased CLT by 6.5% (p = 0.037) when compared with the remainder (Table 3).

**Table 2:** Laboratory investigations

Variable	All subjects (n = 97)	Low polyhedrocytes group (n = 14)	High polyhedrocytes group (n = 83)	p-value
Fibrinogen, g/L	3.31 ± 0.58	2.90 ± 0.46	3.38 ± 0.57	0.004
INR	1.00 (0.96–1.03)	1.02 (0.98–1.06)	0.99 (0.95–1.03)	0.22
aPTT, sec	25.00 (23.95–26.20)	24.75 (24.30–25.10)	25.10 (23.60–26.30)	0.80
WBC, 103/µL	6.93 ± 1.61	7.51 ± 1.62	6.83 ± 1.59	0.15
RBC,106/µL	4.64 (4.31–4.94)	4.62 (4.21–5.29)	4.64 (4.31–4.92)	0.82
HGB, g/dL	13.60 (12.90–14.50)	13.15 (11.98–14.33)	13.60 (13.00–14.50)	0.08
HCT, %	40.20 (38.70–42.90)	39.25 (36.30–42.43)	40.30 (38.70–42.90)	0.30
RDW, %	13.50 (13.00–14.50)	14.80 (13.00–15.90)	13.50 (12.90–14.40)	0.034
PLT 103/µL	204.00 (161.50–249.50)	207.50 (157.50–252.50)	204.00 (166.00–250.00)	0.87
PDW, fL	12.60 (11.40–14.50)	13.20 (11.40–14.80)	12.35 (11.38–14.50)	0.56
Glucose, mmol/L	6.60 (5.90–7.75)	5.70 (5.45–6.83)	6.60 (6.10–7.80)	0.018
HbA1c, %	6.40 (5.93–7.10)	6.50 (5.60–7.08)	6.40 (6.00–7.13)	0.78
Creatinine, µmol/L	82.00 (69.50–96.50)	81.00 (72.25–104.75)	82.00 (68.00–96.00)	0.72
eGFR (mL/min/1.73 m^2^)	76.97 ± 19.10	79.14 ± 22.79	76.60 ± 18.55	0.64
TC, mmol/L	3.93 (3.25–4.74)	3.69 (3.17–4.16)	4.08 (3.26–4.75)	0.30
LDL-C, mmol/L	2.19 (1.67–3.02)	1.79 (1.61–2.80)	2.26 (1.78–3.04)	0.31
HDL-C, mmol/L	1.22 (0.95–1.49)	1.24 (0.83–1.41)	1.22 (0.98–1.53)	0.78
TG, mmol/L	1.33 (1.03–1.73)	1.27 (0.98–2.09)	1.37 (1.03–1.73)	0.67
TSH, mIU/L	1.39 (0.84–2.05)	0.98 (0.88–1.88)	1.48 (0.84–2.12)	0.57
hsCRP, mg/L	2.29 (0.95–4.00)	1.35 (0.97–2.43)	3.02 (0.95–4.23)	0.11

Values are given as mean ± SD or median (interquartile range)

Low polyhedrocytes group represents lowest 10% of the observations. High polyhedrocytes group represents highest 90% of the observations

*aPTT* activated partial thromboplastin time, *eGFR* estimated glomerular filtration rate based on a Modification of Diet in Renal Disease (MDRD) formula, *HDL-C* high-density lipoprotein cholesterol; HGB, hemoglobin, *HCT* hematocrit, *hsCRP* high sensitivity C-reactive protein, *INR* international normalized ratio, *LDL-C* low-density lipoprotein cholesterol, *PDW* platelet distribution width, *RBC* red blood cells, *RDW* red blood cell distribution width, *PLT* platelet count, *TC* total cholesterol, *TG* triglycerides, *TSH* thyroid-stimulating hormone, *WBC* white blood cells, *ACEI* angiotensin-converting-enzyme inhibitors, *ARB* angiotensin receptor blockers, *BMI* body mass index, *MI* myocardial infarction, *T2D* type 2 diabetes

**Table 3:** Coagulation and fibrin clot properties in T2D patients

Variable	All subjects (n = 97)	Low polyhedrocytes group (n = 14)	High polyhedrocytes group (n = 83)	p-value
Lag time, min	2.90 (2.56–3.67)	2.95 (2.54–3.59)	2.81 (2.57–3.67)	0.93
ETP, nM × min	1367.6 (1289.9–1504.8)	1338.7 (1128.0–1380.5)	1382.5 (1291.6–1511.4)	0.15
Peak thrombin generation, nM	264.3 (222.9–309.3)	240.3 (224.4–268.2)	266.0 (222.2–310.9)	0.27
ttPeak, min	5.81 (5.00–7.00)	5.94 (5.48–6.90)	5.67 (5.00–7.05)	0.45
K_s,_ × 10^−9^ cm^2^	5.72 (4.30–6.84)	6.77 (5.56–7.69)	5.56 (4.25–6.57)	0.011
CLT, min	112.0 (100.0–127.0)	108.0 (96.0–114.3)	115.0 (103.0–130.0)	0.037

Values are given as mean ± SD or median (interquartile range)

Low polyhedrocytes group represents lowest 10% of the observations. High polyhedrocytes group represents highest 90% of the observations

*ETP* endogenous thrombin potential, *ttPeak* time to thrombin generation peak, *K*_*s,*_ fibrin clot permeability coefficient, *CLT* clot lysis time

In the whole group polyhedrocytes content was positively correlated with glucose and TC levels (r = 0.24, p = 0.028 and r = 0.22, p = 0.044, respectively), but not with other lipid parameters. Compared with patients with glucose < 6 mmol/L, those with higher glycemia were more prone to form greater amounts of polyhedrocytes (OR = 4.81, 95% CI 1.49–16.40, p = 0.009).

Platelet markers, fibrinolytic proteins and oxidation parameters are presented in Additional file 1: Table S1. The content of polyhedrocytes was positively correlated with P-selectin (r = 0.54, p = 0.0078) and PF4 (r = 0.59, p = 0.0032), but not with platelet count, which was within the reference range for all patients. Of the oxidative stress markers, the content of polyhedrocytes was strongly associated with total PC (r = 0.67, p = 0.0004) and TBARS (r = 0.60, p = 0.0027).

### Compression of erythrocytes

In the HP patients the median of the new parameter describing compression of erythrocytes, ECI was 59.4 (53.7–69.6)%. There were no associations between ECI and demographic or clinical parameters in those patients. ECI was strongly associated with total PC (r = 0.79, p = 0.036) and with fibrinogen level (r = 0.29, p = 0.030).

### Clot composition

The detailed analysis of the blood clot composition (n = 40) showed that their major components were native erythrocytes (32.6 ± 16.6%), transitional erythrocytes (28 ± 12.6%), and fibrin (24.5 ± 9.4%). Polyhedrocytes accounted for nearly 8% and platelets for 1% of clot composition. Interestingly, the percentage of polyhedrocytes was positively associated with HbA_1c_ (r = 0.40, p = 0.024) (Fig. 1).

### The influence of exogenous glucose on polyhedrocyte formation

The in vitro increase of glucose concentration from 4.9 to 14.9 mmol/L in blood obtained from healthy subjects was associated with higher content of polyhedrocytes (36 [19–44] % to 47 [43–55]%; p = 0.044). Similarly in T2D subjects the increase of blood glucose concentration from 7.1 to 17.1 mmol/L was associated with increased content of polyhedrocytes (from 17 [14.5–24.2]% to 33 [25–41.9]%; p = 0.033).

### Predictors of the HP content

After adjustment for age, sex and fibrinogen, multiple logistic regression analysis showed that increasing RDW was the only negative independent predictor of polyhedrocytes content in clots (OR = 0.61, 95% CI 0.39–0.92). Simple linear regression models showed that 1% increase in percentage of polyhedrocytes was significantly associated with the increase in TBARS (by 0.4%), total PC (by 0.5%), P-selectin (by 0.5%), and PF4 (by 0.3%) (Table 4).

**Table 4:** Regression analysis of the influence of the increased polyhedrocytes content in blood clots of T2D (increase by 1%)

Variable	Estimated change of the variable	Standard error	p-value	R^2^ (%)
TBARS, nmol/mL	0.14	0.037	0.0018	41.0
Total PC plasma, nmol/mg	0.012	0.0028	0.0005	48.2
P-selectin, ng/mL	0.13	0.059	0.048	19.0
PF4, ng/mL	0.38	0.15	0.019	25.7

R^2^ represents the coefficient that is a measure of fitting the model

*PF4* platelet factor 4, *TBARS* thiobarbituric acid reactive substances, total PC plasma, total protein carbonyl in plasma

## Discussion

The present study identifies several factors that affect formation of polyhedrocytes in patients with T2D and high cardiovascular risk. Our study is the first to demonstrate that both the glycaemia and HbA_1c_ were associated with increased polyhedrocytes formation in T2D patients, which was confirmed by in vitro experiment. Moreover, increased platelet activation, reflected by plasma concentrations of P-selectin and PF4, and protein oxidation, in particular total PC, were the major determinants of polyhedrocytes formation in blood clots of T2D patients. Our findings indicate that regulation of polyhedrocyte formation in clots is complex and dependent on factors other than RBC, platelet count and fibrinogen concentration.

Moreover, the link between the extent of oxidative stress and polyhedrocytes formation in T2D is also a novel observation. We found that compression of the erythrocytes measured as a new parameter called ECI in patients with T2D was strongly positively associated with protein oxidation. Therefore, we may speculate that disturbed clot contraction associated with oxidative stress in patients with a long-standing history of T2D and high cardiovascular risk could be one of the mechanisms behind increased atherothrombotic complication in those patients. Recently ECI was investigated in patients with venous thromboembolism (VTE) [28]. In contrast to VTE patients in whom ECI was related to age, BMI, platelet count and RDW [28] no such relationships were observed in our patients with T2D. Similarly to previous observations in patients with stroke and VTE clot contraction in T2D was associated with fibrinogen level [28, 34]. It has been shown, that compression of erythrocytes was preferably associated with platelet/fibrinogen ratio rather than the platelet or fibrinogen alone [18, 28, 35]. This may indicate the importance of major platelets effect, which can be modulated by the fibrin, and its influence on the clot contraction [18, 23].

RBCs are especially sensitive cells, therefore they are important health indicators [26]. Assessment of RBCs in terms of biophysical and shape changes such as eryptosis, may be helpful in the evaluation of hematological changes associated with inflammatory status or disease progression [26, 36–38]. We have observed eryptotic cells within clots made from the whole blood obtained from T2D patients (Fig. 1a, marked with arrows).

The clot’s ability to contract is a feature of each patient and could be modulated by many transient factors [21]. The major players in this process are platelets and fibrin [34]. During clot contraction, fibrinogen and fibrin fibers are required as the substrate upon which the aggregating platelets pull, and the fibrin(ogen) connecting the platelets binds to the platelet integrin αIIbβ3. Clot contraction driven by platelet contractile proteins, particularly by the actin-myosin interactions is crucial especially in the first phase of clot contraction [23, 39]. Through keeping a local tension to reestablish the fibrin-fibrin network, the platelet/fibrin ratio was found to play an important role in clot contractility [23]. Furthermore, clot retraction requires energy which is derived from glucose metabolism by platelets and in the absence of glucose, retraction will not occur [40]. In our study, we demonstrated that platelet-derived proteins, i.e. P-selectin and PF4 released from alpha granules upon activation were associated with the increased content of polyhedrocytes in blood clots. This is a novel observation. Growing evidence suggests that oxidative stress can affect platelet reactivity and intensify platelet aggregation process [41]. Therefore, it seems that the oxidative stress can also affect fibrin clot contraction by affecting the platelet reactivity. Nevertheless, platelets in T2D patients are displaying an ‘angry’ behavior. The lysosomal granules might have a significant role in T2D followed by cardiovascular complications [42]. Platelet surface receptors and platelet activation were found to be elevated in T2D, showing increased procoagulant activity and hyperreactivity [42, 43]. These coagulopathies are accompanied by augmented LPS-binding protein (LBP) levels [44].

Tutwiler et al. reported that higher hematocrit along with an increased RBC rigidity were associated with limited clot contractility [23]. In our study, T2D patients who formed more polyhedrocytes within blood clots had similar RBC and hematocrit, but lower RDW. Moreover after the adjustments, lower RDW was the only independent predictor of greater polyhedrocytes formation. It is possible that erythrocytes of similar size are more likely to form polyhedrocytes, while different sizes of RBCs hinder this process. Thus, increased RDW would result in poorer ability of erythrocytes to provide an impermeable seal, due to minimal interstitial space, to increase fibrinolysis resistance. Furthermore, the inflammatory process might play an important role in increased RDW values by releasing the immature RBCs which may contribute to the impaired clot contraction [45]. This issue requires further investigation.

We have shown positive association of polyhedrocytes formation with worse glycemia control. Moreover, in the vitro experiment we demonstrated, that higher glucose concentration is associated with higher polyhedrocyte content in blood clots. Erythrocytes of patients with T2D exhibit impaired deformability related to increased HbA_1c_ and accumulation of intracellular sorbitol or to the stiffness of red blood cell membranes probably due to oxidative modification of the proteins and to an imbalance of the cholesterol/phospholipids ratio [46]. Moreover, RBCs incubated in an elevated level of glucose showed a significantly increased protein glycation and induced ATPases activity [47] and were are highly susceptible to oxidative damage as a result of the high polyunsaturated fatty acid content of their membranes and the high cellular concentration of oxygen and hemoglobin [46]. Therefore, we hypothesize that these processes may impact the rheological properties of RBCs, cause membrane damage and potentially alter the RBCs contraction, influencing blood clot shrinkage properties.

Patients who formed more polyhedrocytes in our study showed also prothrombotic fibrin clot properties, such as decreased K_s_ or prolonged CLT. We have previously demonstrated that prothrombotic fibrin clot phenotype and impaired fibrinolysis might be associated with prolonged T2D duration [48]. Moreover, we have previously shown that in VTE patients, greater ECI (less compression) was associated with prolonged CLT, when compared to controls [28]. Carroll et al. have also shown that clot contraction significantly facilitated clot lysis [22]. The current findings suggest that fibrin features measured in plasma clots generated in vitro that are related to T2D may also be the markers of clot compression.

### Limitations

First, a limited number of patients have been included in the study, therefore subgroup comparisons should be analyzed cautiously. Second, HbA_1c_ was measured using immunoturbidimetry, but not by high-performance liquid chromatography. Third, we did not measure the RBCs membranes rigidity or assess function of platelets such as platelet aggregation. It is unclear whether polyhedrocytes formation affects the risk of cardiovascular events or thrombosis in T2D as suggested by other studies with regard to MI [24] or stroke [34]. Finally, subjects of the present study were all Caucasians, and we did not analyze genetic subtypes of the subjects studied.

## Conclusions

Increased glucose levels, enhanced oxidative stress and platelet activation were associated with increased formation of polyhedrocytes in contracted blood clots. The significance of in vivo polyhedrocytes in T2D and in other diseases remains to be established and further studies are necessary to demonstrate the role of polyhedrocytes in thromboembolic events.

## Additional file

**Additional file 1: Table S1.** Platelet markers, fibrinolytic proteins and oxidation parameters in a subset of the study group T2D patients (n=23).

## Abbreviations

T2D: type 2 diabetes
RBCs: red blood cells
eGFR: estimated glomerular filtration rate
CAD: coronary artery disease
MI: myocardial infarction
SPECT: single photon emission computed tomography with Tc-99 m-MIBI
aPTT: activated partial thromboplastin time
TC: serum total cholesterol
LDL-C: low-density lipoprotein cholesterol
HDL-C: high-density lipoprotein cholesterol
TG: triglycerides
TSH: thyroid stimulating hormone
HbA1c: glycated hemoglobin A1c
RDW: red blood cell distribution width
WBC: white blood cells
SEM: scanning electron microscope
ECI: erythrocyte compression index
CAT: calibrated automated thrombography
TF: tissue factor
Ks: permeation coefficient
rtPA: recombinant tPA
total PC: total protein carbonyl
DNPH: dinitrophenylhydrazine
TCA: trichloracetic acid
TBARS: thiobarbituric acid reactive substances
TAC: total antioxidant capacity
ORAC: oxygen radical absorbance capacity
BIC: Bayesian Information Criterion
OR: odds ratio
CI: confidence interval
LP: low-polyhedrocytes group
HP: high-polyhedrocytes group
IQR: interquartile range
CLT: clot lysis time
VTE: venous thromboembolism
PF-4: platelet factor-4
