# Supplementary material for: Polyhedrocytes in blood clots of type 2 diabetic patients with high cardiovascular risk: association with glycemia, oxidative stress and platelet activation
Source: Cardiovasc Diabetol. 2018 Nov 22;17:146. doi: 10.1186/s12933-018-0789-6 (PMC6251112; doi:10.1186/s12933-018-0789-6)
Supplement: Supplementary file 1 — Additional file 1: Table S1. Platelet markers, fibrinolytic proteins and oxidation parameters in a subset of the study group T2D patients (n=23). [file 12933_2018_789_MOESM1_ESM.docx]

**Additional file**

**Additional Table S1.** Platelet markers, fibrinolytic proteins and oxidation parameters in a subset of the study group T2D patients (n=23).

| **Platelet parameters** | |
| --- | --- |
| P-selectin, ng/mL | 23.87±5.62 |
| PF4, ng/mL | 138.55±15.79 |
| **Fibrinolytic proteins** | |
| α2AP, % | 93.87±18.67 |
| Plasminogen, % | 96.91±14.32 |
| PAI-1, ng/mL | 10.03±3.82 |
| PAI-1 antigen, ng/mL | 26.37 (18.8-29.7) |
| **Oxidation parameters** | |
| TBARS, nmol/mL | 33.48±4.32 |
| TAC, nmol/mL | 245±35.06 |
| Total PC plasma, nmol/mg | 2.46±0.34 |

Values are given as mean ±SD or median (interquartile range).

Abbreviations: α2AP, alpha-2 antiplasmin; au, absorbance units; PAI-1, plasminogen activator inhibitor 1; PF4, platelet factor 4; TAC, Total antioxidant capacity; TBARS, Thiobarbituric Acid Reactive Substances; Total PC plasma, Total Protein Carbonyl in plasma.
